# Efficacy of Curcumin-Mediated Antimicrobial Photodynamic Therapy on *Candida* spp.—A Systematic Review

**DOI:** 10.3390/ijms25158136

**Published:** 2024-07-26

**Authors:** Magdalena Kubizna, Grzegorz Dawiec, Rafał Wiench

**Affiliations:** 1 Department of Oral Surgery, Faculty of Medical Sciences in Zabrze, Medical University of Silesia, 40-055 Katowice, Poland; mkubizna@acstom.bytom.pl (M.K.); gdawiec@acstom.bytom.pl (G.D.); 2 Department of Pediatric Otolaryngology, Head and Neck Surgery, Chair of Pediatric Surgery, Medical University of Silesia, 40-752 Katowice, Poland; 3 Department of Periodontal Diseases and Oral Mucosa Diseases, Faculty of Medical Sciences in Zabrze, Medical University of Silesia, 40-055 Katowice, Poland

**Keywords:** aPDT, *Candida*, diode laser, oral candidiasis, planktonic cells, biofilm

## Abstract

Oral candidiasis is a common problem among immunocompetent patients. The frequent resistance of *Candida* strains to popular antimycotics makes it necessary to look for alternative methods of treatment. The authors conducted a systematic review following the PRISMA 2020 guidelines. The objective of this review was to determine if curcumin-mediated blue light could be considered as an alternative treatment for oral candidiasis. PubMed, Google Scholar, and Cochrane Library databases were searched using a combination of the following keywords: (*Candida* OR candidiasis oral OR candidiasis oral OR denture stomatitis) AND (curcumin OR photodynamic therapy OR apt OR photodynamic antimicrobial chemotherapy OR PACT OR photodynamic inactivation OR PDI). The review included in vitro laboratory studies with *Candida* spp., in vivo animal studies, and randomized control trials (RCTs) involving patients with oral candidiasis or prosthetic stomatitis, published only in English. The method of elimination of *Candida* species in the studies was curcumin-mediated aPDT. A total of 757 studies were identified. Following the analysis of the titles and abstracts of the studies, only 42 studies were selected for in-depth screening, after which 26 were included in this study. All studies evaluated the antifungal efficacy of curcumin-mediated aPDT against *C. albicans* and non-*albicans Candida*. In studies conducted with planktonic cells solutions, seven studies demonstrated complete elimination of *Candida* spp. cells. The remaining studies demonstrated only partial elimination. In all cases, experiments on single-species yeast biofilms demonstrated partial, statistically significant inhibition of cell growth and reduction in biofilm mass. In vivo, curcumin-mediated aPDT has shown good antifungal activity against oral candidiasis also in an animal model. However, its clinical efficacy as a potent therapeutic strategy for oral candidiasis requires few further RCTs.

## 1. Introduction

*Candida* species (*C.* spp.) are commensal yeasts found on various mucosal surfaces and human skin. A properly functioning immune system limits excessive growth [1,2]. However, in immunocompromised individuals, *Candida* spp. can become opportunistic pathogens that cause candidiasis. The development of this disease is influenced by both local and systemic factors. Local factors include the use of dentures, poor oral hygiene, dry mouth, smoking, and long-term use of inhaled corticosteroids. Systemic factors include immunodeficiency (such as Human Immunodeficiency Virus—HIV infection), diabetes mellitus, hematologic diseases (such as leukemia), dysbiosis caused by long-term antibiotic therapy, chemotherapy, use of immunosuppressive drugs after transplantation, and advanced age [3]. Candidiasis is the most prevalent infectious condition of the oral mucosa, and *Candida albicans* is the most frequently isolated species for oral infections [4]. *Candida* spp. possess a range of virulence factors, such as strong adhesion and biofilm formation, which impede drug action and increase resistance to antimicrobial agents. Additionally, they exhibit resistance to host defense mechanisms and oxidative stress, produce tissue-destructive hemolytic enzymes, and develop resistance to previously used antifungal drugs [5]. *Candida* species release proteinases, phospholipases, and acidic metabolites, which damage the structure and function of human cells [6]. The filamentous form (hyphae) of *Candida albicans* invades the oral mucosa epithelium through endocytosis mediated by epithelial cells or active penetration, where a live hypha penetrates through or between cells. This invasion results in necrosis or apoptosis of cells, causing damage to the colonized epithelium [7]. Systemic candidemia can lead to a high mortality rate, especially in severe cases [8]. Additionally, *Candida auris* is a rare and highly resistant strain that poses a significant threat, with resistance rates higher than other *Candida* species. It is known to cause invasive infections in immunocompromised patients, particularly in hospital wards [9]. *Candida* spp. increase the risk of developing oral squamous cell carcinoma by producing carcinogenic substances such as endogenous nitrosamines and acetaldehyde. The infection causes hydrolases and esterases to promote metaplasia and cause changes in the morphology and genotype of cells. These substances are formed from nitrites and nitrates and are direct carcinogens. This predisposes to the development of precancerous conditions and tumors [10].

To effectively treat oral candidiasis, it is crucial to focus on excluding predisposing factors, improving oral and denture hygiene, discontinuing wearing dentures at night, and cushioning or replacing dentures. Local or systemic antifungal therapy is the traditional pharmacological treatment, which includes four main types of antifungal drugs: polyenes, azoles, allylamines, and echinocandins [11]. By following these methods, successful treatment of oral candidiasis can be achieved with confidence. This treatment may cause adverse effects, such as hepatotoxicity in older individuals. However, it is important to note that the response to treatment can be slow and its effectiveness may be low, with a high risk of disease recurrence [12]. Prolonged exposure to medication or repeated cycles of pharmacological treatment can lead to treatment failures due to the development of microorganism resistance. Although nystatin suspension is currently the first-line drug for the treatment of oral candidiasis, it must be used several times a day for an extended period, which often results in drug resistance and various side effects such as abdominal pain, diarrhea, nausea, vomiting, taste disorders, loss of appetite, and irritation of the mucous membrane [13]. Systemic use of fluconazole or amphotericin B is suggested for patients with lowered immunity. It is important to note that azole group drugs have fungistatic rather than fungicidal effects, which may result in insufficiently effective treatment of immunocompromised patients [14,15].

Alternative methods such as the use of plant derivatives in phytotherapy, probiotics that restore the balance of oral microbiota in the presence of dysbiosis, and antimicrobial photodynamic therapy (aPDT) confidently support or replace traditional pharmacotherapy due to the decreasing efficacy of candidiasis treatment and the development of resistant strains. aPDT is a highly selective operation that destroys microorganisms in a nonspecific manner, without any side effects [16]. The treatment has a high antimicrobial potential and is minimally invasive. Unlike conventional treatments, aPDT does not require a high drug dose to be maintained during therapy. Additionally, the development of microbial resistance is unlikely due to the lack of a specific target in the body [17]. APDT is an effective method for promoting cell damage and death in *Candida* support species. This therapy involves combining a photosensitizing agent (PS) and a light source that corresponds to the PS absorption band, which in the presence of oxygen causes the formation of reactive species such as singlet oxygen, superoxide radical, hydroxyl radical, and hydrogen peroxide. These products, as a result of the generated oxidative stress, promote cell damage and death [18]. Yeast cells can protect themselves against oxidative stress due to their adaptive abilities. Their larger size, complex structure, thick cell wall, and nuclear membrane make it difficult for aPDT and PS to penetrate the cell. This reduces the number of singlet oxygen targets per unit volume of the cell, requiring higher concentrations of photosensitizer and light doses to effectively inactivate the yeasts. *Candida* spp. increase the regulation of antioxidants by producing enzymes such as superoxide dismutase and catalase [11].

Curcumin, a natural compound found in turmeric, has been shown to have antioxidant properties. This substance has a wide range of beneficial effects, including anti-inflammatory, antioxidant, anticancer, and antimicrobial properties. It has been shown to alter the integrity of cell membranes and affect membrane-associated proteins [19]. Additionally, it promotes wound healing and epithelial regeneration by stimulating growth factors, activating fibroblasts, promoting granulation tissue formation and collagen synthesis, and neovascularization [20]. Furthermore, it has been demonstrated to effectively absorb blue light. The antifungal effect of curcumin as a PS in photodynamic therapy has been demonstrated to be achieved at 20 µM with light and at 173.73 µM without light [7]. Curcuminoids, including curcumin, demethoxycurcumin, and bisdemethoxycurcumin, have slightly different properties [21]. Bisdemetoxycurcumin binds effectively to bacterial cell walls through hydrogen bonds. However, its disinfectant properties are reduced due to the presence of methoxy groups. It is important to note that curcumin is the strongest antioxidant, followed by demethoxycurcumin and bisdemethoxycurcumin [13]. The clinical application of curcumin is hindered due to its insolubility in water, rapid degradation by hydrolysis, proteolysis, and photodegradation, as well as low bioavailability [22]. However, curcumin can be effectively dissolved in solvents such as 10% dimethyl sulfoxide (DMSO) and ethanol or propylene glycol [23].

This study aimed to evaluate, by a systematic review of in vitro studies, animal studies, and randomized clinical trials, the possibility of eliminating *Candida* spp. causing oral candidiasis using curcumin-mediated aPDT.

## 2. Material and Methods

### 2.1. Focused Question

A systematic review was conducted following the PICO framework, as follows: do *C. albicans* and non-*albicans Candida* strains, that often cause oral candidiasis (Population), subjected to curcumin-mediated aPDT (Intervention) compared to the irradiation of blue light alone, curcumin as PS alone, or other pharmacological treatment methods (Comparison), result in their eradication or more effective elimination (Outcome)?

### 2.2. Information Sources and Search Strategy

The review was conducted following the Preferred Reporting Items for Systematic Reviews and Meta-Analyses (PRISMA 2020) guidelines [24]. An electronic search through PubMed/Medline, Google Scholar, and Cochrane Library databases was conducted. The following medical subject headings (MeSHs), terms, keywords, and their combinations were used: (Candida OR candidiasis oral OR denture stomatitis) AND (photodynamic therapy OR aPDT OR photodynamic antimicrobial chemotherapy OR PACT OR photodynamic inactivation OR PDI OR curcumin). The databases were searched by three authors, each of whom searched separately using the same search terms. The authors then applied additional electronic filters (articles published between 1 January 2007 and 16 January 2024, and only reports published in English). After screening and selecting potential studies for inclusion in the review, all authors jointly assessed the titles and abstracts of the above articles to see if the study met all inclusion criteria. Subsequently, to collate data from the included studies, the authors performed a collaborative search of the full texts to identify the desired data. Additionally, the authors conducted a snowball search to identify further studies. This involved searching the reference lists of publications that were deemed eligible for full-text review.

### 2.3. Study Selection

This study hypothesized that curcumin-mediated aPDT may effectively reduce *Candida* strains and be a supportive or alternative method of treating oral candidiasis to traditional pharmacological methods of treatment. The criteria for the inclusion and exclusion of articles from this review were as follows.

Inclusion criteria:

- in vitro studies involving *C. albicans* or other non-*albicans Candida* stains;
- animal studies involving *C. albicans* or other non-*albicans Candida* stains;
- RCTs involving patients with oral candidiasis or denture stomatitis;
- *Candida* elimination method used in in vitro studies and in animal studies, and RCT was curcumin-mediated aPDT.

Exclusion criteria:

- case reports or case series;
- letters to the editor;
- historic reviews;
- reviews or systematic reviews;
- books and documents;
- duplicated publications or studies with the same ethical approval number;
- studies published in a non-English language;
- general medical applications;
- aPDT form not used as therapy;
- curcumin used not as a photosensitizer;
- other PS than curcumin was used;
- blue light used without PS;
- no *Candida* strains evaluated;
- endodontic, carious, or bone models, not related to oral candidiasis.

### 2.4. Risk of Bias in Individual Studies

In the initial phase of the study selection process, each reviewer individually assessed titles and abstracts to mitigate potential biases in the evaluation process. Cohen’s к test was employed as a tool to quantify the level of inter-reviewer agreement [25]. Any discrepancies regarding the inclusion or exclusion of a study in the review were discussed by the authors until a consensus was reached.

### 2.5. Quality Assessment and Risk of Bias across Studies

Two reviewers (M.K. and R.W.) conducted independent screenings of the included studies to assess their quality. The criteria used to determine the design, implementation, and analysis of the study were based on the presence of key information for the course of aPDT and the objectivity and verification of study results. The risk of bias was determined by the number of “yes” or “no” responses to the questions below that were assigned to each study.

- Was there a specific concentration of photosensitizer?
- Was the origin of the photosensitizer provided?
- Was an incubation time indicated?
- Were the light source parameters provided, such as type, wavelength, output power, fluence, and power density?
- Were clinical strains of *Candida* spp. used in the study?
- Was a negative control group included?
- Were there numerical results (statistics)?
- No missing outcome data?

Additionally, in the case of RCT:

- Did the study include at least 10 patients per group?
- Was there a minimum 6-month follow-up period?

The information collected about the research was assessed. The classification was based on the total number of “yes” answers to the above questions. In the current study, the degree of bias was calculated according to the point limits given below:

(1) High risk: 0–3;
(2) Moderate risk: 4–6;
(3) Low risk: 7–8.

The scores for each study were calculated, and an overall estimated risk of bias (low, moderate, and high) was determined for each included study, following the recommendations outlined in the *Cochrane Handbook for Systematic Reviews of Interventions* [26].

### 2.6. Data Extraction

Having reached a consensus regarding the selection of included articles, the two reviewers (M.K. and G.D.) involved subsequently extracted data regarding:

- citation (first author and publication year);
- type of study;
- type of *Candida* strains used in the study;
- test/control groups;
- follow-up;
- outcomes;
- type and parameters of the light source;
- curcumin concentration;
- use of nanocarriers and additional substances, incubation, and irradiation time.

## 3. Results

### 3.1. Primary Outcome

The primary objective of this systematic review was to assess the efficacy of curcumin-mediated aPDT in the eradication of *Candida* strains responsible for oral candidiasis and to examine the methodologies employed in the studies.

### 3.2. Study Selection during Full-Text Analysis

**Figure 1:**
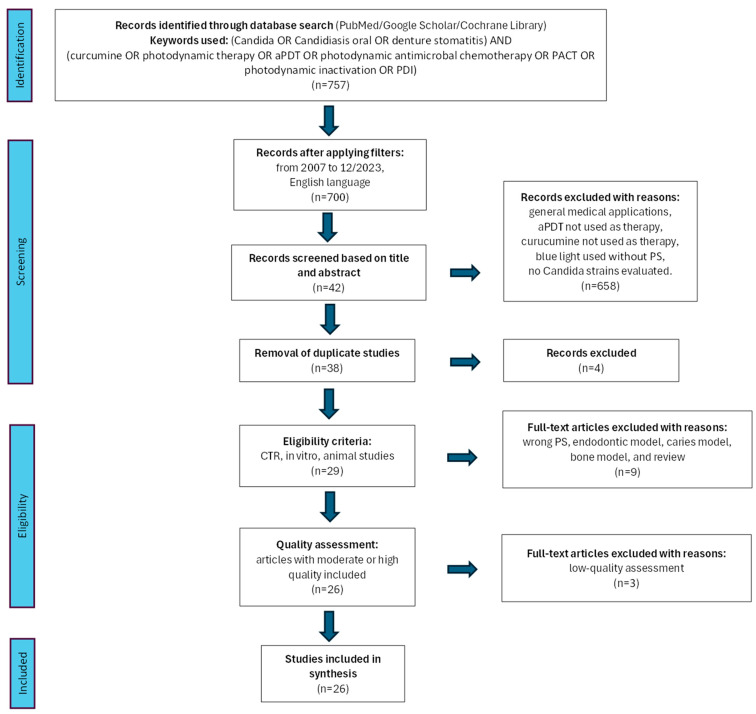
PRISMA 2020 flowchart of selected criteria for the included article reports.

A flowchart representing the research approach following the PRISMA 2020 statement [24] is displayed in Figure 1.

A primary search of the databases yielded 757 articles. After applying the additional electronic filters (published in the period from 1 January 2007 to 16 January 2024 and only English-language reports), the preliminary number of 757 articles was reduced to 700 that were selected for the title and abstract screening. After the screening, 658 studies were excluded as they did not fulfill the inclusion criteria. After the removal of four duplicated articles, a total of 38 studies underwent a comprehensive analysis of their full texts. Nine of these studies were excluded due to failing to meet the predefined exclusion criteria [27,28,29,30,31,32,33,34,35]. Table 1 presents the excluded studies and the reason for their exclusion.

### 3.3. Quality Assessment Presentation

Table 2 presents the risk of bias in 29 studies included after full-text analysis. Only studies that scored at least six points were included in the review. Of the included studies, 22 studies received a low risk of bias (2 studies received a maximum score of 8/8 [14,36]; the remaining 20 studies received a maximum score of 7/8 [5,11,13,15,23,37,38,39,40,41,42,43,44,45,46,47,48,49,50,51]. Seven studies received a moderate risk of bias, with four rated 6/8 [7,52,53,54], and three rated 5/8 [6,55,56]. None of the studies were rated at high risk of bias. Two studies that scored less than six points were excluded from further analysis due to the previously adopted criterion.

### 3.4. Data Presentation

Table 3, Table 4 and Table 5 present the extrapolated data regarding the general characteristics of all 26 studies finally included in the review that fulfilled the eligibility criteria, had characteristics of light sources, and the characteristics of curcumin used as photosensitizers in aPDT protocols.

### 3.5. General Characteristics of the Included Studies

A total of 26 studies were included in the review. Table 3 lists the articles that met the inclusion criteria. Of these, 19 were in vitro laboratory studies [5,11,13,15,23,36,39,41,43,45,46,47,48,49,50,51,52,53,54], 1 was an in vitro study with an additional in situ examination in the oral cavity [42], 3 were in vivo animal studies (a mouse model of oral candidiasis) [7,38,40], and the remaining 3 were randomized controlled trials [14,37,44].

**Table 3:** General characteristics of studies that fulfilled the eligibility criteria.

Reference Number Country Year of Publication	Study Design	*Candida* Species	Study Group Number of Repetitions/Participants Demographic Data Clinical Protocol Data	Outcomes
[5] Brazil 2020	In vitro study on a 96-well plate	Reference strain *C. albicans* ATCC 90028 Biofilm	PS-L-, PS-L + 37.5 J, PS-L + 50 J, PS + L-40J, PS + L-80 J, PS + L + 40/37.5 J, PS + L + 40/50 J, PS + L + 80/37.5 J, PS + L + 80/50 J (n = 6)	aPDT 80/50 J promotes a greater reduction in the expression of *C. albicans* genes associated with adhesion and biofilm formation and genes responsible for oxidative stress.
[7] Brazil 2018	In vivo animal study, tongues of mice infected with *C. albicans*	Reference strain *C. albicans* ATCC 90028	CUR + L+, CUR-L-, CUR + L-, AC + L-, AC + L+, CC + L-, CC + L-, NYS1, NYS4; C free CUR, AC anionic CUR, CC cationic CUR, NYS 100 000 IU 1 and 4 x daily (n = 24)	Free CUR shows a better photodynamic effect than NP-CUR in nanocarriers. APDT with free CUR results in tongue epithelial CK13 and CK14 expression like that observed in healthy mice, which was not observed with NYS.
[11] Brazil, Portugal 2015	In vitro study on a 96-well plate	Reference strain *C. albicans* ATCC 18804 Planktonic cultures	PS + L+, PS + L-, PS-L+, PS-L-, H_2_O_2_ 10 mM (n = 3)	aPDT caused extensive DNA damage to *C. albicans*, which was not effectively repaired due to the inhibition caused by CUR.
[13] Thailand 2021	In vitro study on a 6-well plate	Reference strain *C. albicans* ATCC 10231 Biofilm	CUR + L+ (D10, D20, B10, B20, E110, E220, D10 + E110, D10 + E220, D10 + Ti, D20 + E110, D20 + E220, D20 + Ti, B10 + E110, B10 + E22, B10 + Ti, B20 + E110, B20 + E220, B20 + Ti, E110 + Ti, E220 + Ti, D10 + E110 + Ti, D10 + E220 + Ti, D20 + E110 + Ti, D20 + E220 + Ti, B10 + E110 + Ti, B10 + E220 + Ti, B20 + E110 + TI, B20 + E220 + Ti), NYS CUR-L- (n = 9)	20 µM bisdemethoxycurcumin + erythrosine 110–220 µM + 10% titanium nanoxide tend to generate relatively large amounts of ROS and effectively inhibits *Candida albicans* without inducing cytotoxicity against normal human gingival fibroblasts.
[14] Saudi Arabia 2021	A randomized controlled clinical trial with a 12-week follow-up	Oral candidiasis, prosthetic stomatitis; *C. albicans*, *C. tropicalis*, *C. glabrata*	RB + L+ (15 individuals—3 male and 12 female; mean age 57.2); CUR + L+ (15 individuals—5 male and 10 female; mean age 59.5); NYS (15 individuals—4 male and 11 female; mean age 56.9). Inclusion criteria: completely edentulous participants, removable complete denture wearers diagnosed with denture stomatitis, and habitual cigarette smokers. Random assignment of participants to one of the three research groups. Treated oral mucosa of the palate and denture plate. Six sessions for each participant—thrice per week for a half month.	CUR-mediated aPDT is as effective as topical NYS therapy in treating denture-induced stomatitis in cigarette smokers.
[15] Brazil 2012	In vitro study on a 96-well plate	Reference strain *C. albican*s ATCC 90028, *C. glabrata* ATCC 2001, *C. dubliniensis* CBS 7987 Planktonic cell solutions and biofilms	PS + L+, PS-L+, PS + L-, PS-L- (n = 10)	*C. albicans*—cell viability decreases proportionally regardless of concentration, best effect 20 min PIT and 40 µM CUR. *C. glabrata*—best effect 40 µM CUR, dependence on PIT unclear. *C. dubliniensis*—Groups irradiated for 4 min were concentration-dependent for extreme values (40 and 20 µM). In contrast, groups irradiated for 8 min were concentration- and incubation time-dependent.
[23] Austria 2021	In vitro study in Eppendorf tubes	Reference strain *of C. albicans* ATCC Mya 273 Planktonic form	CUR-L-, CUR-L+, CUR + L-, CUR + L+; 5 µM 0/5/25 min, 10 µM 0/5/25 µM, 20 µM 0/5/25 min, 50 µM 0/5/25 min (DMSO 5/10%) (n = 3)	CUR shows the best antimicrobial activity at a concentration of 50 µM without an incubation period regardless of the DMSO concentration.
[36] Brazil 2017	In vitro study on a 96-well plate	Clinical strain *C. dubliniensis* CD6, CD7, CD8, reference strain CBS 7987 (control) Plankton cultures and biofilms	PS-L+, PS + L-5, PS + L-10, PS + L-20, PS-L-, PS + L + 5, PS + L + 10, PS + L + 20 (for planctonic forms); PS-L+, PS + L-20, PS + L-30, PS + L-40, PS-L-, PS + L + 20, PS + L + 30, PS + L + 40 (for biofilms) (n = 15)	The best therapeutic effect against plankton forms CUR 20 µM and against biofilms CUR 40 µM.
[37] Brazil 2022	Controlled, two-arm, parallel-group, single-blind clinical trial	Clinical strain, oral candidiasis, C. tropicalis, *C. parapsilosis*, *C. krusei*, *C. glabrata*	MB—39 patients (33 male and 6 female) between 40 and 83 years (mean = 61.49); CUR—37 patients (27 male and 10 female) between 34 and 77 years (mean = 60.11) Inclusion criteria: individuals with a confirmed histopathological diagnosis of SCC in the oral cavity, larynx, oropharynx, nasopharynx, or hypopharynx treated with RT and presented with oral candidiasis. Treated was the entire oral lesion surface. The aPDT session was twice a week for two weeks.	Curcumin at 80 µmol/L irradiated with an energy of 200 J/cm^2^ is associated with increased free radical generation. CUR was less effective than TBO.
[38] Brazil 2023	In vivo study in a mice model of oral candidiasis	Reference strain *C. albicans* ATCC 90028	CUR + L + 20, CUR + L + 40, CUR + L + 80, CUR + L-20, CUR + L-40, CUR + L-80, CUR-L+, CUR-L- (n = 5)	Histological analysis of the tongues of mice treated with aPDT 80 µM CUR showed a reduced number of *Candida* cells that were confined to the stratum corneum and low inflammatory response.
[39] Brazil 2020	In vitro test on silicone samples in a 24-well plates	Reference strain *C. albicans* ATCC 90028 Biofilm	L-CUR-, L-CUR+, L + CUR-, L + CUR+ (n = 2)	The antimicrobial effect on *C. albicans* depends on the concentration of curcumin and the exposure time. The best results are obtained with 60 µg/mL curcumin and 30 min of irradiation.
[40] Brazil 2012	In vivo study in a mice model of oral candidiasis	Reference strain *C. albicans* ATCC 90028	PS + L + 20 µM, PS + L + 40 µM, PS + L + 80 µM PS + L-20 µM, PS + L-40 µM, PS + L-80 µM, PS-L+, PS-L- (n = 5)	A curcumin concentration of 80 uM combined with LED light causes the greatest change in the number of *C. albicans* colonies.
[41] Brazil 2017	In vitro study on a 96-well plate	Reference strain *C. albicans* ATCC 90028 Biofilm, planktonic cultures	CUR + L+ (free CUR, anionic CUR, cationic CUR), CUR + L-, CUR-L+, CUR-L- NL- (anionic and cationic nanoparticles without CUR) (n = 6)—anionic CUR, cationic CUR (n = 3)—free CUR	Anionic CUR shows the lowest antibacterial photodynamic effect; cationic CUR was cytotoxic.
[42] Brazil 2021	In vitro study on a 96-well plate. In situ biofilm study in the oral cavity (volunteers wore palatal appliances containing enamel samples to establish dental biofilms in situ; study on a 24-well plate)	Reference strain *C. albicans* ATCC 90028 Biofilm	CUR + L+ (CUR-LCP, CUR-CHIH, CUR-ME, CUR-S), CUR + L-, CUR-L+, CUR-L- (n = 12)—in vitro study (n = 7)—in situ biofilm study	CUR-S is the only formulation that can significantly reduce the viability of the biofilm after photodynamic treatment.
[43] Brazil 2022	In vitro study on a 24-well plate	Reference strain *C. albicans* ATCC 18804, *C. tropicalis* ATCC 13803 Planktonic cultures	CUR-L- (saline), N (nystatin), C. longa + L-, CUR + L-, CUR-L+, CUR + L+ (n = 10)	The isolated curcumin longa extract and photodynamic therapy with CUR have antifungal activity against *C. albicans* and *C. tropicalis* and no toxicity to the invertebrate model *G. mellonella*.
[44] Saudi Arabia 2023	Randomized controlled clinical trial with 2-month follow-up	Prosthetic stomatitis; *C. albicans*, *C. krusei*	Group I (antifungal gel therapy)—25 patients (9 male and 16 female), mean age 55.2; 5 blocks/study group; every block comprised 5 subjects; Group II (aPDT CUR + antifungal gel)—25 patients (10 male and 15 female), mean age 56.7; 5 blocks/study group; every block comprised five subjects Inclusion criteria: edentulous patients using complete dentures. Treated was the oral mucosa of the palate and denture plate. The PDT over four weeks and eight weeks, twice a week with a 48 h interval between each session.	CUR-mediated aPDT is an effective treatment method for reducing the mycological burden on the palate mucosa and denture surfaces, as well as improving salivary pro-inflammatory cytokine levels in patients with denture-related stomatitis.
[45] Brazil 2011	In vitro study on a 96-well plate	Reference strain *C. albicans* ATCC 90028 Planktonic cultures, biofilm	PS + L+, PS + L-, PS-L-, PS-L+ (n = 5)	The highest therapeutic efficacy 20 µM CUR, 5.28 J/cm^2^, 20 min incubation time.
[46] Brazil, Netherlands 2021	In vitro study on a 96-well plate	Reference strain of *C. albicans* ATCC 18804 Planktonic forms and biofilm	PS-L-, PS-L+, CHX, NYS, PS-D + L-, PS-D + L+, PS + D + L-, PS + D + L+, PS-M + L-, PS-M + L+, PS + M + L-, PS + M + L+ (n = 9)	CUR-Plu shows a lower reduction than CUR-DMSO. Multispecies biofilm shows greater resistance than monospecies. CUR-Plu can be considered a stable and effective method for controlling biofilm within a short time after synthesis.
[47] Brazil 2011	In vitro study on a 96-well plate	Clinical isolates *C. albicans* Ca1, Ca2, Ca3, Ca4, Ca5; *C. glabrata* Cg1, Cg2, Cg3, Cg4, Cg5; *C. tropicails* Ct1, Ct2, Ct3, Ct4, Ct5 Planktonic cell solution and biofilm	PS + L+, PS + L-, PS-L+, PS-L- (n = 5)	The greatest reduction in the activity of *C. albicans*, *C. tropicalis*, and *C. glabrata* using 40 µM CUR and 18 J/cm^2^.
[48] Brazil 2023	In vitro study on a 96-well plate	Reference strain *C. albicans* ATCC 90028 Planktonic cultures and biofilms	CUR + L+, CUR + L-, CUR-L+, CUR-L- (n = 12)	*C. albicans* planktonic cultures are susceptible to subsequent applications of sublethal aPDT doses via CUR. Sublethal aPDT CUR may have made *C. albicans* cells more resistant to therapy.
[49] Brazil 2023	In vitro study on a 96-well plate	Reference strain *C. albicans* SC 5314 Planktonic cultures and biofilms	CUR-L-, F + CUR-L-, P + CUR-L-, M + CUR-L-, F + CUR + L-, P + CUR + L-, M + CUR + L-, CUR + L-, CUR-UL+, CUR-BL+, M + CUR-UL+, M + CUR + UL+, M + CUR + BL+, F + CUR + BL+, P + CUR + BL+, CUR + BL+, M + CUR + UL + BL+ (n = 12)	CUR-loaded F127 micelles bound to blue light caused biofilm photoinactivation.
[50] Brazil 2016	In vitro test on acrylic samples in a 24-well plate	Reference strain *C. albicans* ATCC 90028, *C. glabrata* ATCC 2001 Biofilm	PS + L + (24 h), PS + L-(24 h), PS + L + (48 h), PS + L-(48 h), PS-L + (24 h), PS-L + (48 h), PS-L-(24 h), PS-L-(48 h) (n = 9)	The 24 and 48 h biofilms are susceptible to CUR-mediated aPDT at concentrations assessed at 37.5 J/cm^2^.
[51] Thailand 2023	In vitro study on a 6-well plate	Reference strain *C. albicans* ATCC 10231 Biofilm	CUR20 + L+, CUR40 + L+, CUR80 + L+, KI + L+, CUR20 + KI + L+, CUR40 + KI + L+, CUR80 + KI + L+, nystatin, phosphate-buffered saline (n = 5)	A mixture of 40 uM bisdemethoxycurcumin + 100 mM KI combined with blue light can effectively reduce *C. albicans* biofilm after 6 h with efficacy comparable to NYS.
[52] Saudi Arabia, Australia 2017	In vitro test on Petri dishes	Reference strain *C. albicans* ATCC 10231 Cells in water mixture, cells on agar surface	PS + L+, PS + L-	*C. albicans* in an aqueous mixture are inhibited at any light dose, on the agar surface at 96 J/cm^2^, spore soaking had no significant effect on cell number reduction.
[53] Taiwan 2018	In vitro study on a 96-well plate	Reference strain *C. albicans* ATCC 90029 Planktonic cell solutions, adherent cultures	PS + L+, PS + L+ 208 µM fluconazole (n = 3)	Fluconazole eliminates the yeast form, CUR-aPDT the biofilm. Fluconazole + CUR-aPDT eliminate the growth and virulence of *C. albicans.*
[54] Italy, France 2017	In vitro test on solid medium plates and in Eppendorf tubes	Reference strain *C. albicans* SC 5314 Planktonic cell solution, adherent cultures	PS + L+, PS + L-, PS-L+, PS-L- (n = 2)	100% inhibition of *C. albicans* growth at any fluence.

**Abbreviations:** L—light, PS—photosensitizer, CUR—curcumin, C.—*Candida*, ACTT—American Type Culture Collection, h—hours, AC—anionic curcumin, CC—cationic curcumin, NYS—nystatin, MB—methylene blue, CUR—curcumin, N—anionic and cationic nanoparticles without curcumin, DMSO—dimethylsulfoxide, CUR-LCP—liquid crystalline precursor system with curcumin, CUR-CHIH—chitosan hydrogel with curcumin, CUR-ME—microemulsion with curcumin, CUR-S—free curcumin dissolved in 10% DMSO, D—demetoxycurcumin, B—bisdemetoxycurcumin, E—erythrosine, Ti—titanium nanoxide, CHX—chlorhexidine, M—micelles, F—F127, P—P123, UL—ultraviolet, BL—blue light, KI—potassium iodide, RB—Rose Bengal, SCC—squamous cell carcinoma, RT—radiotherapy, n—number of **repetitions/participants**.

The materials tested in vitro included planktonic cell solutions (three studies) [11,23,43], single-species biofilms (six studies) [5,13,39,42,50,51], and planktonic cultures and biofilms (eleven studies) [15,36,41,45,46,47,48,49,52,53,54]. Animal models were employed, including a mouse model of oral candidiasis after prior pharmacological immunosuppression (prednisolone + tetracycline) [7,38,40].

In vitro studies with planktonic cell solutions were conducted in 96-well [5,11,15,36,41,42,45,46,47,48,49,53], 24-well [39,42,43,50], and 6-well [13,51] titration plates; Eppendorf tubes [23,54]; or Petri dishes [52]. Biofilm studies were conducted on silicone [39], acrylic [50], or Sabouraud dextrose agar [52].

The Candida strains that were tested included Candida albicans (twenty-four tests) [5,7,11,13,14,15,23,38,39,40,41,42,43,44,45,46,47,48,49,50,51,52,53,54], Candida krusei (two tests) [37,44], Candida glabrata (five tests) [14,15,37,47,50], Candida tropicalis (four tests) [14,37,43,47], Candida duliniensis (two tests) [15,36], and Candida parapsilosis (one test) [37]. The majority of the studies employed reference strains from the American Type Culture Collection, with five studies utilizing clinical strains (obtained from patients with oral candidiasis) [14,36,37,44,47]. Nineteen studies were conducted exclusively on Candida spp. strains, while eight studies were additionally tested: Escherichia coli [39], Staphylococcus aureus MRSA [39,41,49], Streptococcus mutans [41,42,46,50], Aspergillus niger [23,52], Aspergillus flavus [52], Lactobacillus casei [42], Penicillinum griseofulvum [52], Penicillinum chrysogenum [52], Fusarium oxysporum [52], Zygosaccharomyces bailii [52], and Pseudomonas aeruginosa [49].

The experimental groups in the studies analyzed typically included the following treatments: (PS + L+) light irradiation in the presence of a photosensitizer; (PS-L+) light-only treatment without a photosensitizer; and (PS + L-) application of only a photosensitizer without the presence of light. The negative control group (PS-L-) consisted of leaving the planktonic cell solution or biofilm in the dark without exposure to light or photosensitizer (curcumin).

### 3.6. Characteristics of Light Sources Used in aPDT

The characteristics of the physical parameters of the light sources that met the inclusion criteria are presented in Table 4.

**Table 4:** Physical parameters of light sources from studies that met the eligibility criteria.

Reference Number	Light Source	Wavelength (nm)	Energy Density (Fluence) (J/cm^2^)	Power Output (mW)	Irradiation Time	Spot Size/Fiber Surface Area (cm^2^)
[5]	LED	450	37.5, 50	30	21, 27 min	-
[7]	LED (LXHL-PR09, Luxeon III Emitter, Lumileds Lighting, San Jose, CA, USA)	455 (440–460)	37.5	75	7 min	0.196
[11]	LED (LXHL-PR09, Luxeon III Emitter, Lumileds Lighting, San Jose, CA, USA)	455 (440–460)	37.5	89.2	7 min	0.196
[13]	Dental lamp (VALO Ortho Cordless, South Jordan, UT, USA)	395–480	72	3200	27 s	0.747
[14]	LED (LXHL-PR09, Luxeon III Emitter, Lumileds Lighting, San Jose, CA, USA)	455 (440–460)	37.5 (denture) 122 (palate)	260	26 min (denture), 20 min (palate)	0.196
[15]	LED (Institute of Physics Sao Carlos, SP, Brazil)	455	5.28	22	4 min	-
[23]	LED self-made	435	15.8	-	60 min	-
[36]	LED (LXHL-PR09, Luxeon III Emitter, Lumileds Lighting, San Jose, CA, USA)	455 (440–460)	5.28	22	4 min	0.196
[37]	Blue LED (New Dent s/n)	480	200	480	90 s	0.216
[38]	LED (University Sao Paulo, Sao Carlos, SP, Brazil)	455	37.5	89.2	7 min	-
[39]	LED (Biotable RGB, MMOptics, Sao Carlos, SP, Brazil)	430	10.8, 32.4	18	10, 30 min	-
[40]	LED (LXHL-PR09, Luxeon III Emitter, Lumileds Lighting, San Jose, CA, USA)	455 (440–460)	37.5	89.2	7 min	0.196
[41]	LED (LXHL-PR09, Luxeon III Emitter, Lumileds Lighting, San Jose, CA, USA)	455 (440–460)	43.2	33.58	20 min	0.196
[42]	LED (LXHL-PR09, Luxeon III Emitter, Lumileds Lighting, San Jose, CA, USA)	450 (440–460)	18	22	14 min	0.196
[43]	Prototype device based on LEDs (Biotable Irrad/LED)	450 ± 5	10, 25	110	91, 228 s	-
[44]	LED (LXHL-PR09, Luxeon III Emitter, Lumileds Lighting, San Jose, CA, USA)	455 (440–460)	37,5 (denture) 122 (palate)	260	26 min (denture), 20 min (palate)	0.196
[45]	LED (LXHL-PR09, Luxeon III Emitter, Lumileds Lighting, San Jose, CA, USA)	455 (440–460)	37.5, 1.32, 2.64, 3.96, 5.28, 6.60, 13.20, 26.40	22	29 min, 1, 2, 3, 4, 5, 10, 20 min	0.196
[46]	LED (Biotable RGB, MMOptics, Sao Carlos, SP, Brazil)	460	15	-	11 min 36 s	-
[47]	LED (LXHL-PR09, Luxeon III Emitter, Lumileds Lighting, San Jose, CA, USA)	455 (440–460)	5.28, 18, 25.5, 37.5	22	n.a	0.196
[48]	LED (Biotable 3.4, Sao Carlos, Brazil)	450	18	47	6.38 min	-
[49]	LED (Biotable RGB, MMOptics, Sao Carlos, SP, Brazil)	455	33.84	47	12 min	-
[50]	LED (LXHL-PR09, Luxeon III Emitter, Lumileds Lighting, San Jose, CA, USA)	455 (440–460)	37.5	22	29 min	0.196
[51]	Dental lamp (Elipar DeepCure-L Curing Light, 3M, Singapore)	450 ± 30	90	950	95 s	0.785
[52]	Xenon arc lamp (Polilight, PL 500, Rofin Australia Pty Ltd., Victoria, Australia)	370–680; 420	24, 48, 72, 96, 240, 360	500,000	2, 4, 6, 8, 20, 30 min	-
[53]	LED self-made	430	9	-	30 min	-
[54]	Diode laser blue-violet	405	10, 20, 30	-	50, 100, 150 s	0.2

**Abbreviations:** LED—light-emitting diode.

In the 22 papers analyzed, a light-emitting diode (LED) was used as the light source, with thirteen studies having a maximum emission peak of 455 nm [7,11,14,15,36,38,40,41,44,45,47,49,50]; five studies 450 nm [5,42,43,48,51]; two studies 430 nm [39,53]; and single studies 435 nm [23], 460 nm [46], and 480 nm [37]. The output power of the light sources ranged from 18 to 480 mW, and the fluences used were in the range of 1.32–200 J/cm^2^.

In one study, a 405 nm diode laser was used to perform aPDT [54].

Two studies used dental polymerization lamps with a wavelength of 395–480 nm [13,51]. One study used a 500 W xenon arc lamp equipped with an optical fiber in the 370–680 nm range (420 nm wavelength was chosen for irradiation) [52].

### 3.7. Characterization of Curcumin Used as a Photosensitizer in aPDT

Characteristics of curcumin used as a photosensitizer in aPDT in studies that met the inclusion criteria are shown in Table 5.

**Table 5:** Characteristics of curcumin used as a PS in studies meeting eligibility criteria.

Reference Number	Incubation Time (in Minutes)	The Way of Presentation of Curcumin	Concentration/s of PS Used
[5]	20	CUR (Sigma-Aldrich, St. Louis, MO, USA) CUR diluted in 1% dimethylsulfoxide	40, 80 µM
[7]	20	Free CUR prepared in 10% dimethylsulfoxide diluted in sterile milli-Q water; anionic and cationic curcumin-loaded polymeric nanoparticles	260 µM
[11]	20	CUR (Sigma-Aldrich, St. Louis, MO, USA) A stock CUR solution (600 mM) prepared in 10% dimethylsulfoxide diluted in saline	2, 5 µM
[13]	15	Demethoxycurcumin and bisdemetoxycurcumin (Sigma-Aldrich, St. Louis, MO, USA) powder dissolved in methanol and then dissolved in water to form 200 µM stock solutions	10, 20 µM
[14]	30	CUR (Sigma-Aldrich, St. Louis, MO, USA) powder prepared with phosphate-buffered saline to obtain original concentrations of 5 µg/mL	5 µg/mL
[15]	1, 5, 10, 20	CUR (Sigma-Aldrich, St. Louis, MO, USA) prepared with 10% dimethylsulfoxide to obtain original stock solution	5, 10, 20, 30, 40 µM
[23]	0, 5, 25	The stock solution (100 mM) of curcumin (Carl Roth, Karlsruhe, >90.6%) prepared by dissolving in dimethylsulfoxide; A stock solution (5 nM) of SACUR-12a (Institiute of Organic Chemistry, University of Regensburg, Regensburg, Germany) prepared in ddH_2_O	5, 10, 20, 50 µM CUR + 5% DMSO/10% DMSO; 10, 50, 100 µM SA-CUR12a
[36]	20	CUR (Sigma-Aldrich, St. Louis, MO, USA) prepared with 10% dimethylsulfoxide to obtain an original stock solution	5, 10, 20 µM (for planktonic form), 20, 30, 40 µM (for biofilms)
[37]	1	Curcumin (96% turmeric, Millenium Farmacias Ltd.a., Janauba, Minas Gerais, Brazil) prepared by diluting 0.03 g in 10 mL of dimethylsulfoxide and then adding more 990 mL of ultrapure water	80 µM
[38]	20	CUR-based water-soluble salt mixture (PDTPharma, Cravinhos, Brazil) A water-soluble mixture of curcuminoids prepared in ultra-pure water	20, 40, 80 µM
[39]	5	CUR turmeric powder, lot C1386, content ≥ 65% (Sigma-Aldrich, St. Louis, MO, USA). The excipients used for preparation of the mouthwash: propylene glycol, polyethylene glycol 400, sorbitol, polysorbate 80 or Tween 80, and ultrapure water	30, 60 µg/mL
[40]	20	A stock solution (800 µM) of natural CUR (Sigma-Aldrich, St. Louis, MO, USA) prepared in 10% dimethylsulfoxide and then diluted in saline solution	20, 40, 80 µM
[41]	40	CUR purity ≥ 65% (Sigma-Aldrich, St. Louis, MO, USA) used as a PS in ratio 9:1 of sterile distilled water/DMSO	130 µM
[42]	5	CUR (Sigma-Aldrich, St. Louis, MO, USA) incorporated into three different bioadhesive formulations (CUR-LCP, CUR-CHIH, and CUR-ME); Free CUR solubilized into 10% dimethylsulfoxide and high-purity water (CUR-S)	20, 40, 60, 80 µM
[43]	20	The glycolic extract of C. longa (Seiva Brazilis, Sao Paulo, SP, Brazil) obtained commercially at a concentration of 20% (200 mg/mL); CUR (PDT Pharma, Cravinhos, SP, Brazil) diluted in a 1% dimethylsulfoxide solution and ethanol p.a. and then was kept as a stock solution, posteriorly diluted in phosphate-buffered solution	100 mg/mL CUR longa/200 µg/mL CUR
[44]	30	CUR powder mixed with phosphate-buffered saline for obtaining the original concentration of 5 µg/mL	5 µg
[45]	5, 20	CUR (Sigma-Aldrich, St. Louis, MO, USA) A stock solution of curcumin (200 µM) prepared in 10% dimethylsulfoxide and then diluted in saline solution to obtain the concentrations to be tested	0.005; 0.01; 0.05; 0.1; 0.5; 1; 5; 10; 20 µM
[46]	5	CUR (Sigma-Aldrich, St. Louis, MO, USA, lot number: SLBR4883) disolved in dimethylsulfoxide to obtain the stock solution and then diluted in milli-Q water to obtain the concentrations to be tested	270 µM
[47]	20	CUR (Sigma-Aldrich, St. Louis, MO, USA) A stock solution of curcumin (600 µM) prepared in dimethylsulfoxide and then diluted in saline solution to obtain the concentrations to be tested	5, 10, 20 µM
[48]	20	A solution of 368.38 µM CUR (Sigma-Aldrich, St. Louis, MO, USA) (>65% pure) prepared in 2.5% dimethylsulfoxide and diluted in phosphate-buffered saline to obtain the concentrations to be tested	40 µM
[49]	20	CUR (368.38 g/M, ≥65% purity) (Sigma-Aldrich, St. Louis, MO, USA)—synthesis of photo-responsive micelles	63 µM
[50]	20	CUR (Sigma-Aldrich, St. Louis, MO, USA) A stock solution of curucmin (800 µM) disolved in 10% dimethylsulfoxide and then diluted in physiological solution (0.85% NaCl) to the final concentrations	80, 100, 120 µM
[51]	20	Bisdemetoxycurcumin (MedChem Express, Suite Q, NJ, USA) dissolved in absolute ethanol (final concentration, <1% ethanol) and heated at 70 °C for 5 min to prepare a stock solution	20, 40, 80 µM bisdemethoxycurcumin + 100 µM KI
[52]	10, 20, 30	CUR (Sigma-Aldrich, St. Louis, MO, USA) A stock solution (2000 µM, pH = 4.9) of curcumin powder disolved 73.8 mg in 30 mL of propylene glycol (99.5%) and then diluted in 70 mL of sterile water to obtain the concentrations to be tested	100–1000 µM, 800 µM
[53]	20	Curcumin solutions	1, 5, 10, 20, 40, 80 µM
[54]	n.a.	CUR (Sigma-Aldrich, S.r.l., Milan, Italy) (content ≤ 100%) distilled in dimethylsulfoxide to obtain 20 mM stock solutions	100 µM

**Abbreviations:** CUR—curcumin, DMSO—dimethyl sulfoxide, KI—potassium iodide, PS—photosensitizer, CUR-LCP—liquid crystalline precursor system with curcumin, CUR-CHIH—chitosan hydrogel with curcumin, CUR-ME—microemulsion with curcumin, CUR-S—free curcumin dissolved in 10% DMSO, NaCl—sodium chloride.

In 21 studies, curcumin was the sole photosensitizer utilized [7,11,13,15,23,36,38,39,40,41,42,43,44,45,46,47,49,50,51,52,53]. In five studies, the efficacy of aPDT with curcumin was compared with other photosensitizers, including methylene blue (one study) [37], photodithazine (two studies) [5,48], Rose Bengal (one study) [14], toluidine blue (one study) [54], and erythrosine (one study) [54]. In two studies, the efficacy of curcumin-mediated aPDT was further enhanced by the addition of titanium nanodioxide or erythrosine (one study) [13] or potassium iodide (one study) [51]. A total of eighteen studies employed free curcumin [5,48], while in seven studies, curcumin was incorporated into previously synthesized formulations, including nanocarriers, nanoparticles, micelles (five studies) [7,23,41,46,49], a microemulsion (one study) [39], or a bioadhesive formula (one study) [42]. Two studies used curcumin derivatives: bisdemethoxycurcumin and demethoxycurcumin [13,51]. Two studies combined antimicrobial photodynamic therapy with pharmacotherapy: fluconazole (one study) [53] and a commercial antifungal gel—miconazole (one study) [44].

The incubation times employed in the studies were empirically derived, were derived by the authors based on previous studies by other authors, or were previously employed by the authors as a preliminary test. The incubation times used in the studies ranged from 1 min to 40 min. The most frequently used incubation times were 20 min (sixteen studies) [5,7,11,15,36,38,40,43,45,47,48,49,50,51,52,53] and 5 min (six studies) [15,23,39,42,45,46]. In two studies, the incubation times used were not reported [54,56].

The photosensitizer added to the planktonic cells solution or biofilm was kept in the dark, in a darkened room, or under an adhesive dressing until irradiation in all evaluated studies. During the incubation time, in all studies using planktonic solutions, care was taken to thoroughly mix curcumin with the solution.

## 4. Discussion

All studies that met the inclusion criteria for the review showed significant efficacy of curcumin-mediated aPDT against *Candida* spp. (efficacy is understood as a reduction in cell number or a reduction in % CFU/mL). In studies conducted with planktonic yeast cell solutions, seven studies showed complete elimination of *Candida* spp. cells. [15,45,47,48,52,53,54] and the others showed a partial, statistically significant reduction compared to the control group. Similarly, in all cases of experiments performed on monocellular yeast biofilms, only partial growth inhibition and mass reduction were observed after curcumin-mediated aPDT.

In in vivo studies on a mouse model of candidiasis, a statistically significant elimination of yeast from the mucosa, compared to the control group, was observed [7,38]. In a study by Dovigo et al., the results even showed complete elimination of *C. albicans* from the lingual mucosa in test mice after a single therapeutic intervention (LED 455 nm, fluence 37.5 J/cm^2^, power density 89.2 mW, and curcumin concentration 80 µM) [40]. The therapy was independent of the stage of biofilm development. There was a reduction in the number of yeasts and hyphae in the biofilm [7]. Curcumin-mediated aPDT also promoted a reduction in the expression of *C. albicans* genes related to adhesion and biofilm formation and genes responsible for the oxidative stress response [5].

In clinical trials with patients, curcumin-mediated aPDT was effective in reducing the number of *Candida* spp. cells. [14,37,44]. A clinical study by Labban et al. found aPDT to be as effective as topical nystatin therapy—oral rinsing with a nystatin suspension at a concentration of 100,000 UI/mL for 60 s, four times a day, for 15 days (no statistical significance between study groups) [14]. Another study by Al-Ghamdi et al. confirmed a reduction in the mycological burden on the mucosa of the prosthetic field compared with conventional therapy with an antifungal gel containing miconazole (2% Daktarin Gel Oral, Janssen-Cilag Pharmaceutica, Belgium) and a reduction in the levels of the pro-inflammatory cytokines IL-6 and MMP-8 in the saliva of patients with prosthetic stomatitis [44].

In studies comparing the efficacy of curcumin-mediated aPDT on different yeast strains, a better effect was observed for *C. albicans* compared to other non-*albicans Candida* strains [14,15,43,47,50]. Elimination of the clinical strain *C. glabrata* required higher concentrations of curcumin than *C. albicans* and *C. tropicalis*, yet no eradication of *C. glabrata* was observed, in contrast to *C. albicans* and *C. tropicalis* [47]. In addition, reference strains showing resistance to fluconazole and clinical strains obtained from patients with oral candidiasis showed sensitivity to curcumin-mediated aPDT [14,36,37,44,47]. However, this was lower than the reference strains, which did not show antifungal drug resistance. In a study by Sanita et al., no differences in the efficacy of photodynamic therapy between the clinical and reference strains were found for *C. dubliniensis* [36]. Combined pharmacological treatment with fluconazole at a concentration of 208 µM for 24 h together with photodynamic therapy using 20 µM curcumin and 430 nm light at a fluence of 9 J/cm^2^ was able to significantly reduce up to 5% of *C. albicans* biofilm cell viability when pharmacological monotherapy or photodynamic therapy alone was not sufficiently effective [53]. Experiments on animal models [7,38,40] also confirmed the reduced adhesion of *C. albicans* to epithelial cells and their ability to penetrate deep into the epithelium (reduced thigmotropism). Yeast cells, on microscopic examination, were only detected in the stratum corneum of the epithelium on the dorsal surface of the tongue [38,40]. In addition, histopathological examination of the epithelium and its lining after aPDT treatment showed only a small number of inflammatory cells in the tissue, which suggested, according to the authors, that the treatment did not exacerbate the inflammatory response of the host tissues [7,38]. Curcumin-mediated aPDT also showed no cytotoxicity to human gingival fibroblasts [13] and to the invertebrate model *G. mellonella* [43,54].

The efficacy of antimicrobial photodynamic therapy is strongly dependent on many variable parameters of the algorithm, hence the large discrepancy in results obtained by authors using different therapeutic protocols. One of the key elements is the correct selection of the appropriate wavelength of the light source. In photodynamic therapy, it is extremely important that the electromagnetic wavelength of the light used is matched as closely as possible to the maximum absorption of the PS used. Curcumin shows maximum absorption at 430 nm. In Table 3, in the column describing the wavelength of the light source used in the experiment, it is clear that there are large differences in this parameter. A diode laser with a wavelength of 405 nm [54] and LEDs with a maximum light emission of 430–480 nm were used in 22 studies [5,7,11,14,15,23,36,37,38,39,40,41,42,43,44,45,46,47,48,49,50,53]. However, the most used wavelength was 455 nm [7,11,14,15,36,38,40,41,44,45,47,49,50].

Also, blue light alone, without curcumin, shows fungicidal activity against *Candida* spp. (CUR-L+). It stimulates naturally occurring endogenous photosensitizers in pathogen cells and subsequently leads to the production of cytotoxic oxidative forms. [57,58]. Using the same fluence of blue light as aPDT, however, the efficacy is much lower than when combined with a photosensitizer. In the Jordao et al. study, treatment with blue LED alone (35.7 J/cm^2^ and 50 J/cm^2^) promoted a reduction in the expression of *C. albicans* genes responsible for biofilm formation [5]. Also, a study by Carmello et al. showed that blue LED promoted DNA damage to *C. albicans* [11]. In contrast, in a study by Dias et al., there was a reduction in both planktonic cell solution and biofilm cells in groups treated with light alone [48].

The second important variable element of the aPDT algorithm is the incubation time used (the time between the addition of PS to, for example, the planktonic solution and the start of irradiation—an extremely important time—as it is needed for the right amount of PS to reach the cell membrane and cytoplasm of the yeast cells). In a study by Schamberger et al. comparing the efficacy of photodynamic therapy applied with different incubation times, it was shown that shorter incubation times resulted in a more pronounced increase in aPDT efficacy against yeast in planktonic solution, indicating the limited stability of curcumin in aqueous solution [23]. Curcumin at a concentration of 50 µM irradiated immediately after being injected into solution (incubation time—30 s) showed strong antimicrobial activity. The use of a five-minute incubation time resulted in a decrease in the efficacy of curcumin-mediated aPDT. Increasing the incubation time to 25 min further enhanced this effect [23]. A completely different result was shown in a study by Andrade et al. In a planktonic solution, the use of different incubation times (1, 5, 10, and 20 min), while keeping the light source settings constant, showed no statistical differences in the elimination efficacy of *C. albicans*, *C. glabrata,* and *C. dubliniensis* [15].

The combination of 20 µM curcumin and LED light (fluence 5.28 J/cm^2^ and power density 22 mW/cm^2^) after 5, 10, and 20 min incubation times, promoted the complete elimination of yeast cells. Therefore, the conclusion was drawn that long incubation times are not required for yeast elimination using curcumin-mediated aPDT. In addition, in the same study, the penetration of curcumin deep into biofilms was also checked at different incubation times. Biofilms after 5 and 20 minutes of incubation with 40 µM curcumin were observed with a Leica TCS SPE confocal microscope (Leica Microsystems GmbH, Wetzlar, Germany) in fluorescence mode, using an excitation wavelength of 405 nm and green fluorescence (emission from 450 to 600 nm). Despite the bright green fluorescence observed after 5 min of incubation, brighter fluorescence was observed after a 20 min incubation period. A significantly deeper penetration of curcumin after 20 min into monoculture biofilms was observed than after 5 min. Therefore, longer incubation times have been recommended for biofilm elimination [15]. A study by Al-Asmari et al. confirmed that incubating yeast cells with photosensitizer for 10, 20, and 30 min before irradiation showed no significant difference in their reduction [52].

The concentration of the PS used in antibacterial photodynamic therapy against *Candida* spp. is of paramount importance. Due to the large size of yeast cells (approximately 25–50 times larger than bacteria) and the more complex cell structure (thick cell wall and the presence of a cell nucleus separated from the cytoplasm by a nuclear membrane), a higher concentration of curcumin is required than for the elimination of bacteria [59]. Also, curcumin concentrations used for biofilm destruction must be higher than for planktonic solutions, mainly due to the thickness and complex structure of the biofilm [60]. Several studies have comparatively used different concentrations of photosensitizer [5,15,23,36,38,40,45,47,50,53]. The concentrations of curcumin that obtained the best fungicidal effect in these studies were determined to be 80 µM [5,38,40,50,53], 40 µM [15,36,47], 20 µM [36,45], 60 µM [39], and 50 µM [23]. The proposed differences can be explained by the different effects of PS concentration on *Candida* spp. planktonic solutions and monocellular biofilm structures.

In none of the analyzed biofilm studies was complete elimination of *Candida* spp. cells in this structure achieved at any of the curcumin concentrations tested.

In a study by Dovigo et al., on a mouse model of oral candidiasis, the application of 20, 40, or 80 µM curcumin with 455 nm light (fluence 37.5 J/cm^2^, power density 89.2 mW, and incubation time 7 min) was able to cause a statistically significant reduction in the number of viable *Candida* cells compared to untreated mice. However, only a concentration of 80 µM curcumin led to the complete elimination of *C. albicans* from the mucosa of mouse tongues [40].

The use of a photosensitizer on nanocarriers or as a micellar solution [7,41,46,49] required higher concentrations of curcumin. In two studies, the highest concentration of curcumin evaluated was 260 µM (higher concentrations caused curcumin aggregation) [7,41].

Also, curcumin itself has been shown to exhibit anticancer, antioxidant, anti-inflammatory, and antimicrobial activities and to promote wound healing [61]. As a natural product, curcumin has been extensively studied as a drug candidate and has been reported to exhibit antifungal activity against *Candida* spp. [62]. In a study by Sanita et al., exposure to curcumin alone at the highest concentrations used (30 µM and 40 µM) significantly reduced the viability of the *C. dubliensis* biofilm compared to the control group. In contrast, in the study by Dias et al. in the C + L- (40 µM) groups, there was a reduction in both planktonic forms and *C. albicans* biofilm cells [48]. Curcumin at lower concentrations showed no inhibitory effect on *Candida* spp. Sublethal doses of curcumin showed antifungal effects only when activated with light.

Another very important element influencing the efficiency of the photodynamic response is the physical parameters of the light source, expressed in particular as energy density (fluence). Six studies have compared the effects of different fluences on the efficiency of curcumin-mediated aPDT [5,39,45,47,52,54]. In several of these, complete elimination of planktonic forms of *C. albicans* was achieved. In the study by Dovigo et al., the most effective and complete elimination of planktonic forms of *C. albicans* occurred at a fluence of 37.5 J/cm^2^ (455 nm), combined with a curcumin concentration of 20 µM. Furthermore, the minimum fluence causing complete yeast inactivation in this study was 5.28 J/cm^2^ [45]. In contrast, a study by Merigo et al. reported 100% inhibition of yeast growth in a planktonic solution using 405 nm diode laser light, with fluences of 10, 20, and 30 J/cm^2^) [54]. Comparing the results of all the studies in which complete eradication of *C. albicans* in planktonic solution was achieved [15,45,47,48,53,54], the fluences used ranged from 5.28 to 18 J/cm^2^, and these were lower values than when acting with photodynamic therapy on biofilm structures.

In studies in which curcumin-mediated aPDT was directed against *Candida* spp. biofilms, the applied fluences were higher than in studies conducted on planktonic forms. Applied fluences ranged from 10.8 to 96 J/cm^2^ [5,13,39,41,42,46,49,50,51,52]. However, no study achieved complete elimination of yeast in the biofilm. In a study by Jordao et al., a *C. albicans* strain was subjected to LED irradiation (450 nm). At a fluence of 50 J/cm^2^, the greatest reduction in the expression of *C. albicans* genes responsible for biofilm formation and adhesion and for the oxidative stress response was observed [5]. The most promising results of photodynamic therapy in in vivo studies were observed in another study by Dovigo et al. in an animal model using 80 µM curcumin and 37.5 J/cm^2^ fluence irradiation [40]. In a clinical study by Fonseca et al., it was observed that curcumin with a concentration of 80 µM, irradiated with 480 nm light at a fluence of 200 J/cm^2^, generated a higher amount of free radicals, which is associated with better biofilm elimination [37].

In some of the studies reviewed, additional chemicals were used as additives to increase the efficacy of curcumin-mediated aPDT. In two studies in which curcumin derivatives were used, erythrosine, titanium nano-oxide, or potassium iodide were added to increase the effectiveness of the therapy [13,51]. In the study by Kanpittaya et al., the greatest reductions in biofilm composed of *C. albicans* cells were achieved using 20 µM bisdemethoxycurcumin in combination with 110 µM erythrosine and when 20 µM bisdemethoxycurcumin was used with 110 µM erythrosine and 10% titanium nano-oxide. These PS with additives achieved more fungicidal efficacy than pure forms of curcumin derivatives [13]. In a study by Damrongrungruang et al., a therapy combining 40 µM bisdemethoxycurcumin and 110 mM potassium iodide applied for 1 h showed the greatest reduction in *C. albicans* biofilm comparable to 1:100,000 IU/mL nystatin therapy [51].

Two clinical trials reported the use of curcumin-mediated aPDT as an adjunctive treatment to pharmacotherapy [44,53]. The clinical study by Al-Ghambi et al. evaluated the treatment of oral candidiasis with an antifungal gel containing miconazole supported by antimicrobial photodynamic therapy [44]. During the 2-month intervention and follow-up of individual patients, all participants were instructed on denture and oral hygiene. In the control group, patients were instructed to apply a topical antifungal gel, hold it in the mouth for 60 s, and then spit it out. The procedure was repeated four times a day for 15 days. However, in the study group, curcumin-mediated aPDT was added to the antifungal gel treatment protocol. The denture surface and palate were sprayed with 5 mL of PS and then irradiated for 26 min after a 30 min incubation before irradiation. Curcumin-mediated aPDT was carried out for 4 weeks and 8 weeks, twice a week with a 48 h break between each session. Participants in the study undergoing combination therapy showed a significant reduction in *C. albicans* and *C. krusei* abundance compared to the group treated with antifungal gel alone [44]. A study by Hsieh et al. on the effect of the combined drug and photodynamic therapy on *C. albicans* biofilm showed that neither aPDT treatment (reduction to 15%) nor fluconazole monotherapy (reduction to 55% after 24 h and to 20% after 48 h) was sufficiently effective to completely eradicate the infection. In contrast, two treatment protocols: treatment with 208 µM fluconazole for 24 h followed by single aPDT with 20 µM curcumin (fluence 9 J/cm^2^) and treatment with 208 µM fluconazole for 48 h followed by aPDT with 10 µM curcumin (fluence 9 J/cm^2^) reduced yeast cells to 5% [53].

Five studies compared curcumin with other photosensitizers used in aPDT to eliminate *Candida* spp. [5,14,37,48,54]. Studies by Jordao et al. and Dias et al. showed that phenothiazine was as effective as curcumin in inactivating yeast [5,48]. Rose Bengal [14], erythrosine, and toluidine blue [54] are less effective against *Candida* spp., while methylene blue [37] showed a stronger effect. The above studies suggest that curcumin is one of the more effective photosensitizers currently used in aPDT for the treatment of oral candidiasis.

## 5. Conclusions

Curcumin-mediated aPDT against *Candida* spp. may be an effective alternative form of therapy for oral candidiasis associated with restorations. It appears to be particularly useful when dealing with strains resistant to conventional antifungal agents, which is proving increasingly common in clinical practice. APDT can also be used in combination therapy with conventional drug treatment, resulting in much more effective destruction of yeast than monotherapies used. A limitation of aPDT is that it can only treat superficial, localized areas that are accessible for irradiation. The aPDT protocol often requires several minutes of PS incubation before irradiation, which is difficult to achieve in the oral cavity due to saliva flow, among other reasons. Therefore, attempts are being made to place curcumin in nanocarriers to increase curcumin stability or in adhesive formulations to facilitate application. Despite many laboratory studies, optimal PS concentrations and physical parameters of light sources have not been established, which translates into a lack of a recommended clinical protocol.

In available studies in vivo and mouse models of oral candidiasis, curcumin-mediated aPDT shows effective antifungal activity, against *Candida* spp. species causing oral candidiasis. However, the clinical efficacy of aPDT as an effective therapeutic method for the treatment of fungal oral infections requires further study.

## Figures and Tables

**Table 1:** List of excluded studies and the rationale for their exclusion.

Ordinal Number	Reason for Exclusion	Reference Number
1	Review	[27]
2	Endodontic model	[28]
3	Cytotoxicity testing PDT	[29]
4	Endodontic model	[30]
5	Caries model	[31]
6	Bone defect model	[32]
7	Caries model	[33]
8	Caries model	[34]
9	Caries model	[35]

**Table 2:** Risk of bias of 29 studies included for full-text analysis.

Reference Number	PS Concentration	Origin of PS	Incubation Time	Light Source Parameters	Clinical Strains of *Candida* Spp.	Negative Control Group	Numerical Results Available (Statistics)	No Missing Outcome Data	10 Patients per Group	6-Month Follow-Up Period	Total Score 8/2 (RCT)
[5]	yes	yes	yes	yes	no	yes	yes	yes	-	-	7/-
[6]	yes	yes	no	yes	no	yes	yes	no	-	-	5/-
[7]	yes	no	yes	yes	no	yes	yes	yes	-	-	6/-
[11]	yes	yes	yes	yes	no	yes	yes	yes	-	-	7/-
[13]	yes	yes	yes	yes	no	yes	yes	yes	-	-	7/-
[14]	yes	yes	yes	yes	yes	yes	yes	yes	yes	no	8/1
[15]	yes	yes	yes	yes	no	yes	yes	yes	-	-	7/-
[23]	yes	yes	yes	yes	no	yes	yes	yes	-	-	7/-
[36]	yes	yes	yes	yes	yes	yes	yes	yes	-	-	8/-
[37]	yes	yes	yes	yes	yes	no	yes	yes	yes	no	7/1
[38]	yes	yes	yes	yes	no	yes	yes	yes	-	-	7/-
[39]	yes	yes	yes	yes	no	yes	yes	yes	-	-	7/-
[40]	yes	yes	yes	yes	no	yes	yes	yes	-	-	7/-
[41]	yes	yes	yes	yes	no	yes	yes	yes	-	-	7/-
[42]	yes	yes	yes	yes	no	yes	yes	yes	-	-	7/-
[43]	yes	yes	yes	yes	no	yes	yes	yes	-	-	7/-
[44]	yes	no	yes	yes	yes	yes	yes	yes	yes	no	7/1
[45]	yes	yes	yes	yes	no	yes	yes	yes	-	-	7/-
[46]	yes	yes	yes	yes	no	yes	yes	yes	-	-	7/-
[47]	yes	yes	yes	yes	yes	yes	yes	no	-	-	7/-
[48]	yes	yes	yes	yes	no	yes	yes	yes	-	-	7/-
[49]	yes	yes	yes	yes	no	yes	yes	yes	-	-	7/-
[50]	yes	yes	yes	yes	no	yes	yes	yes	-	-	7/-
[51]	yes	yes	yes	yes	no	yes	yes	yes	-	-	7/-
[52]	yes	yes	yes	yes	no	no	yes	yes	-	-	6/-
[53]	yes	no	yes	yes	no	yes	yes	yes	-	-	6/-
[54]	yes	yes	no	yes	no	yes	yes	yes	-	-	6/-
[55]	no	yes	no	yes	yes	yes	yes	no	-	-	5/-
[56]	yes	yes	no	yes	yes	yes	yes	no	-	-	5/-

**Abbreviations:** PS—photosensitizer; RCT—randomized controlled trial.
